# Analysis of the Bacterial Biocenosis of Activated Sludge Treated with Leachate from Municipal Landfills

**DOI:** 10.3390/ijerph19031801

**Published:** 2022-02-05

**Authors:** Aleksandra Wdowczyk, Agata Szymańska-Pulikowska, Magdalena Domańska

**Affiliations:** Institute of Environmental Engineering, Wrocław University of Environmental and Life Sciences, pl. Grunwaldzki 24, 50-363 Wrocław, Poland; agata.szymanska-pulikowska@upwr.edu.pl (A.S.-P.); magdalena.domanska@upwr.edu.pl (M.D.)

**Keywords:** bacterial biocenosis, activated sludge, landfill leachate, treatment, fluorescent staining

## Abstract

The influx of toxic pollutants into wastewater treatment plants can negatively affect the quality of the activated sludge (AS). One source is landfill leachate. The identification of microorganisms present in AS is very important, e.g., while improving wastewater treatment technology. Therefore, the aim of the study was to investigate the effect of raw leachate and after purification of Phragmites australis and Ceratophyllum demersum on the composition of the AS bacterial biocenosis. In addition, AS status was assessed by LIVE/DEAD BacLight ™ fluorescent staining. The obtained results showed that the leachate did not significantly affect the cell membranes of AS bacteria, and even a slight improvement was noted. The research carried out using the next-generation sequencing method shows that the origin of the samples (active and closed storage) and the method of processing do not significantly affect the composition of the AS bacterial biocenosis at higher taxonomic levels. However, at the species level, the appearance of bacteria not previously present in AS was observed, namely: Flavobacterium luticocti, Candidimonas nitroreducens and Nitrobacter hamburgensis. The obtained results suggest that the leachate may be a source of microorganisms positively influencing the condition of AS bacteria.

## 1. Introduction

Waste disposal in many countries is still based on landfilling. The formation of leachate is one of the many environmental hazards associated with waste disposal [1]. The composition of leachate is dynamic and changes over time. Therefore, the choice of an appropriate process for its treatment is one of the main difficulties related to leachate management [2,3].

According to the regulations in force in Poland, leachate is classified as industrial wastewater, which requires treatment to a level that depends on a type of final receiver before discharge. Leachate can be transported to municipal wastewater treatment plants, treated or sub-treated on site or recirculated to a waste heap [4,5]. Various biological, physical and chemical methods are used to treat leachate [5,6]. Biological processes are widely used worldwide for the treatment of raw leachate and/or a mixture of leachate and domestic wastewater [7]. Their particular effectiveness is observed in the treatment of leachate from young landfills containing easily biodegradable organic matter [7,8].

Depending on the oxygenation level, biological processes can be divided into: aerobic, hypoxic or anaerobic [9]. Basic technologies used in biological processes include: the Bardenpho (Bernard denitrification phosphorus removal) system with modifications, the UCT (University of Cape Town) system and the A/O (anaerobic/oxic) system often also called Phoredox (phosphorus reduction oxidation). Sequencing biological reactors (SBR), membrane bioreactors (MBR) or anaerobic UASB type reactors are also used [10,11]

Activated sludge is one of the most popular and widely used methods for biological treatment of leachate and/or leachate mixtures with domestic wastewater [12,13]. It is a complex biological structure containing a variety of bacteria, protozoa, periphytes, threadworms, rotifers, fungi, algae, viruses and metazoa. This ecosystem is dominated by bacteria, usually accounting for about 95% of the total number of microorganisms contained in activated sludge [14]. The dominant type is Proteobacteria, which usually account for 21% to 65% of the bacteria present in activated sludge, further subordinate types present in sludge include Bacteroidetes, Acidobacteria and Chloroflexi [15]. 

An important role in wastewater treatment plants is attributed to the bacteria that carry out the basic process of biological nitrogen removal i.e., nitrification. Nitrification was conventionally classified as a two-stage process. In the first stage, bacteria oxidize ammonium nitrogen (N-NH_4_) to nitrite nitrogen (N-NO_2_) (ammonia oxidizing bacteria -AOB), the second stage is carried out by bacteria (NOB) oxidizing nitrite nitrogen (N-NO_2_) to nitrate nitrogen (N-NO_3_) [16]. From a phylogenetic perspective, five types of AOB bacteria are classified as Nitrosomonas, Nitrosolobus, Nitrosovibrio, Nitrosospira and Nitrosococcus and as NOB bacteria as Nitrobacter, Nitrospina and Nitrococcus [17]. However, this division into two groups was challenged when a new nitrification process, commamox (COMplete AMMonium OXidation) was discovered by bacteria of the genus Nitrospira [18], which are capable of carrying out both stages of nitrification, i.e., they encode oxidation pathways for both ammonia and nitrite [19].

The structure of bacterial communities occurring in activated sludge is affected by many factors, including the source of wastewater and its physicochemical properties (e.g., pH, macronutrient content, presence of organic pollutants or heavy metals) [20,21].

In addition to traditional methods, molecular methods are used to identify microorganisms. In traditional methods, bacterial cultures are isolated and then identified based on their properties. Due to the limitations of these methods, the PCR (Polymerase Chain Reaction) method and its modifications are currently most commonly used for identification of microorganisms: RT-PCR (Reverse Transcriptase Polymerase Chain Reaction), qPCR (Quantitive Polymerase Chain Reaction), a technique using restriction fragment length polymorphism—RFLP (Restriction Fragment Length Polymorphism), or Next-generation sequencing (NGS) with a rich gene library, which has gained popularity in recent years [22]. These methods provide new information on the structure of microbial communities and allow for greater precision than traditional methods [23,24]. 

The identification of microorganisms present in activated sludge is very important, among other things, for the improvement of wastewater treatment technology or selection of optimal solutions in case of problems [15]. 

The operation of a municipal wastewater treatment plant can be disturbed e.g., by the supply of industrial wastewater, which can adversely affect the quality of activated sludge [25]. One possibility of disturbances occurring in the activated sludge process is sludge swelling, which results from excessive growth of filamentous forms of bacteria (filamentous swelling), or a decrease in cohesion and floc density (non-filamentous swelling) [26,27]. In addition to sludge swelling, sedimentation problems can include dispersive microbial growth and an increase in the number of free-floating bacteria, foaming and sludge flowing out as a result of excessive denitrification [25].

Due to the variety of substances present in leachate, difficulties in treatment are very often encountered. Biological treatment of leachate on site, e.g., in constructed wetlands (CW), may not give as good an effect as in the case of domestic wastewater [28] but may provide means of preparation for further treatment in a municipal wastewater treatment plant.

So far, few studies have been conducted on the composition of the bacterial biocenosis of activated sludge and leachate from municipal landfills. Most of the studies conducted have focused only on the analysis of the composition of the activated sludge bacterial biocenosis itself, while there are no studies that would verify the effect of leachate on biocenosis AS.

It is suspected that the time-varying amount of leachate generated by landfills and the excessive content of organic components, as well as their potential toxicity, may disturb the biological balance of the activated sludge biocenosis [29].

Therefore, the present study aimed to evaluate the influence of landfill leachate (raw and after treatment with *P. australis* and *C. demersum*) on the composition and state of the bacterial biocenosis of activated sludge. 

The exact conditions of the experiment conducted on the effectiveness of leachate treatment by *P. australis* and *C. demersum* are presented in an earlier publication [30].

The following were analysed:

✓ physicochemical properties of leachate from an active closed municipal waste landfill,
✓ leachate toxicity (by means of toxicity tests on *D. magna* aquatic organisms),
✓ bacterial biocenosis composition (by next-generation sequencing (NGS, with particular emphasis on nitrifiers) and activated sludge condition (by LIVE/DEAD BacLight™ fluorescent staining)—raw and landfill leachate treated.

## 2. Materials and Methods

Studies on the physicochemical composition and toxicity of leachates from municipal waste landfills in Poland were conducted in 2018–2020 (8 series of studies in total). 

### 2.1. Leachate Samples

Two municipal waste landfills, located in Lower Silesia Voivodship, at different stages of operation, were selected for the study. The first one, not exploited, is located in Bielawa (geographical coordinates 51°9′21.485″ N, 17°14′18.03″ E). The facility was in operation since 2001, for a period of 10 years. Its area is 0.86 ha and its capacity is 37.8 thousand m^3^. 

The second storage site is located in Legnica (geographical coordinates 51°14′21.317″ N, 16°11′0.251″ E) and has been exploited since 1977. Its area is 14.12 ha and its total capacity is 2.34 million m^3^. 

Activated sludge for testing was collected in August 2020 from Janówek (geographical coordinates 51°11′41.0″ N, 16°54′35.8″ E) wastewater treatment plant, located in Wrocław. It is a mechanical-biological treatment plant with chemical support for the removal of phosphorus compounds and full sludge management. Its capacity is 140,000 m^3^/d. 

**Figure 1:**
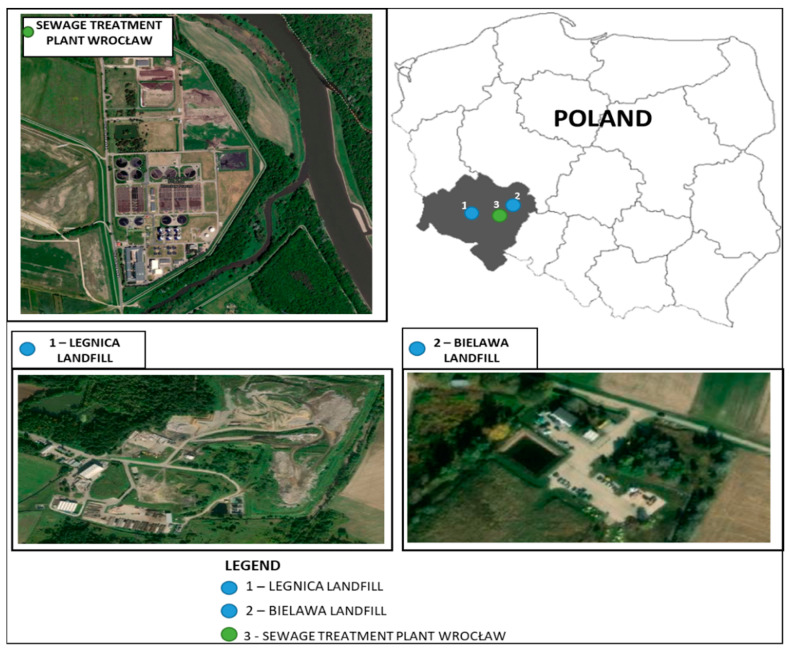
Facilities on which investigations were conducted [geoportal.gov.pl].

Figure 1 presents objects on which investigations were conducted.

### 2.2. Physicochemical Composition of Leachate and Activated Sludge

Immediately after sampling, the samples were transported to the Environmental Research Laboratory of the Institute of Environmental Engineering, Wrocław University of Environmental Sciences. The analyses of physicochemical properties were carried out by means of commonly used methods in accordance with ISO (International Organization for Standardization) standards. Laboratory analyses not requiring mineralisation of samples were performed within 24 h of sample collection [31]. At the same time, mineralisation was carried out, followed by analyses requiring mineralisation.

Raw and treated leachate were tested for: pH, electrical conductivity (EC), Chemical Oxygen Demand (COD), biochemical oxygen demand and concentration (BOD5), total Kjeldahl nitrogen (TKN), organic nitrogen (ON), ammonium nitrogen (AN), total phosphorus (TP), total solids (TS), total dissolved solids (TDS), total suspended solids (TSS), sulphates, chlorides, sodium, potassium, calcium, magnesium, iron, manganese, zinc, lead and nickel, cadmium.

Upon arrival in the laboratory, the activated sludge was analysed for pH, EC, ammonium nitrogen (N-NH_4_^+^), nitrite nitrogen (N-NO_2_^−^) and nitrate nitrogen (N-NO_3_^−^). 

Table 1 presents a list of applied methods of analysis of physicochemical parameters.

### 2.3. Organisation and Conduct of the Experiment

The mixture of leachate feeding the activated sludge test system consisted of raw leachate from two municipal waste landfills (collected on 20 June 2020) and leachate after biological treatment (with *P. australis* and *C. demersum*).

(1) Raw leachate

The raw leachate after collection was transported to the laboratory where it was subjected to physicochemical composition analyses and toxicity tests. Part of the raw leachate was frozen at −18 degrees C until use. One day before the actual experiment, raw leachate samples were thawed with aeration.

(2) Leachate after biological treatment with *P. australis* and *C. demersum*

After 14 days of acclimatisation to laboratory conditions, selected *P. australis* seedlings were transferred individually to 1.5 dm^3^ containers, while *C. demersum* plants were transferred, with two plants to each 0.5 dm^3^ container. The containers were then filled with landfill leachate. Exposure to leachate lasted another 14 days [35,36,37]. No additional aeration was applied during the experiment. Each variant was performed in triplicate. The study included evaluation of the effectiveness of leachate treatment by *P. australis* and *C. demersum* using physicochemical analyses and toxicity tests. The exact conditions of the conducted experiment were presented in an earlier publication [30].

(3) Activated sludge

On the day of the experiment, activated sludge was collected from Janówek municipal wastewater treatment plant located in Wrocław and transported under refrigerated conditions to the laboratory. 

The actual experiment was conducted using activated sludge, raw leachate and leachate after biological treatment with *P. australis* and *C. demersum*.

In 5 dm^3^ reactors, landfill leachate and activated sludge were placed in equal proportions (1:1). The experiment was conducted in tanks with continuous aeration, hydraulic retention time (HRT) was 24 h [38]. The control sample was activated sludge.

**Figure 2:**
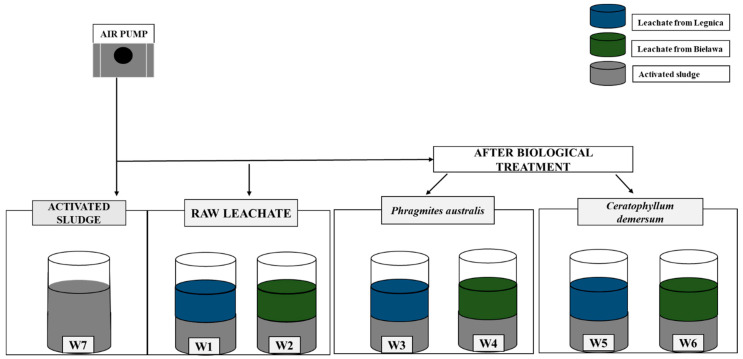
Scheme of conducted experiment with activated sludge mixed with: raw leachate samples from Legnica (W1) and Bielawa (W2), leachate samples after treatment with *P. australis* from Legnica (W3) and Bielawa (W4), leachate samples after treatment with *C. demersum* from Legnica (W5) and Bielawa (W6). W7—control sample (activated sludge).

Figure 2 shows a schematic of the experiment conducted. The experiment was aimed at checking the influence of landfill leachate (raw and after treatment with *P. australis* and *C. demersum*) on the composition and condition of activated sludge bacterial biocenosis.

### 2.4. Acute Toxicity Test on Freshwater Crustacean Daphnia magna Straus

Toxicity tests on *D. magna* crustaceans were performed in accordance with ISO 6341:2012. They were conducted based on the concentration range established when performing the preliminary tests. The test consisted of preparing the following solutions of the test sample: 100%, 50%, 25%, 12.5%, 6.3% and 3.2%. A similar range of sample concentrations (6.25, 12.5, 25, 50, 100%) was used in other acute leachate toxicity tests [39].

Dechlorinated tap water was used to dilute the leachate, which also served as the control sample. In each replicate, five organisms were subjected to the toxicity test. The initial test was carried out in one replicate (five organisms), the specific test included four replicates (five each, total 20 organisms) for each dilution.

In each prepared test solution of 0.1 dm^3^ volume, five organisms of *D. magna* were placed for 24 h. After 24 h exposure, the degree of immobilisation of organisms exposed to each test solution was visually assessed. The toxic effect was expressed as the average percentage of immobilisation of individuals. Immobile organisms were considered to be those that were unable to swim for 15 s after shaking the sample, even if they could still move their antennae. When the number of immobile organisms in a control sample was more than 10%, the control sample tested was considered unrepresentative.

Acute toxicity to *D.magna* organisms is defined as the average concentration that causes an immobilisation effect in 50% of the organisms tested. Based on the analyses performed, EC50 values were determined, determining the leachate concentration that caused immobilisation effect in 50% of the tested organisms. Based on the EC50 values, the TU (Toxic Unit) value was calculated according to the formula:

(1) TU=(1EC50)·10

According to the TU value, leachate toxicity can be divided into:

− no acute toxicity: TU < 0.4,
− slight acute toxicity: 0.4 < TU < 1,
− acute toxicity: 1.0 < TU < 10,
− high acute toxicity: 10 < TU < 100,
− very high acute toxicity: TU > 100 [40,41,42].

### 2.5. Next Generation NGS Sequencing and Fluorescent Staining

DNA was extracted from activated sludge samples in three technical replicates using the GeneMatrix Environmental DNA/RNA Extraction kit (Eurx, Gdańsk, Poland) according to the manufacturer’s instructions. 

The quality of the obtained material was checked by electrophoresis in 1% agarose gel. In order to assess the amount of matrix supplied, measurements were also performed using a Qubit 3.0 fluorimeter (Thermo Scientific) and a dedicated Qubit High Sensitivity DNA kit reagent.

The choice of primers for sequencing follows the recommendations presented in the literature [43].

A 200 mg sample of the mixture was taken for DNA extraction. Several 5 × 5 mm pieces of filter after the filtration of these samples were used for the final analyses.

Sequencing of all samples and controls was performed on the Illumina MiSeq sequencing system. Specific primers were used to amplify the 16SrRNA gene fragment in the samples.

The amplification reaction was performed in an ABI 9700 thermocycler (Life Technologies) using the thermostable polymerase Kapa HiFi PCR Mix (Roche). A library for high-throughput sequencing was prepared, the sequencing reads obtained were filtered and low-quality reads were removed, and species composition analysis was performed on the samples.

The amplification reaction of the bacterial 16SrRNA gene fragment (V3–V4) was performed by the DNA Sequencing and Oligonucleotide Synthesis Laboratory, Institute of Biochemistry and Biophysics, PAS, Warsaw, Poland. Analysis of the obtained data on the composition of the bacterial biocenosis of the activated sludge was performed using the EzBioCloud service https://www.ezbiocloud.net/, accessed on 31 October 2020) and the 16S-based MTP Microbiome Taxonomic Profiling tool [44].

Data generated and used in this study, Illumina, were deposited in the NCBI SRA databases under the bioproject accession number PRJNA801220.

The LIVE/DEAD BacLight™ fluorescence staining method, which determines cell viability based on cytoplasmic membrane continuity, was used to assess the physiological status of activated sludge bacterial cells subjected to landfill leachate stress.

Use of the LIVE/DEAD^®^ kit enables identification of viable and damaged bacterial cells. The kit contains two dyes: the green-fluorescent nucleic acid dye SYTO 9 and the red-fluorescent nucleic acid dye, propidium iodide (PI). Live bacteria are stained with SYTO 9 (green) and damaged bacteria are stained with PI (red) [45]. Samples were stained according to the manufacturer’s instructions of Thermo Fisher Scientific.

### 2.6. Data Treatment and Statistical Analysis

The obtained results were statistically analysed using Statistica 13.1 program (StatSoft Polska, StatSoft, Inc., Tulsa, OK, USA). Basic non-parametric statistics (minimum, maximum) were used to characterise selected physicochemical properties and toxicity of leachates, used among others at small sample size [46]. Principal component analysis (PCA) was used to indicate the factors affecting the values of the studied variables to the greatest extent and to indicate the cases (taxonomic level units) most related to the principal components [31,47].

## 3. Results and Discussion

### 3.1. Selected Physicochemical Properties of Leachate and Activated Sludge

Table 2 presents the results of physicochemical analyses of raw leachates from landfills, collected in June 2020, which were used in the conducted experiment to determine the impact on the biocenosis of activated sludge. These were compared with the results of analyses carried out in 2018–2019 (seven series of tests). As part of the study of the physicochemical composition of leachates, a total of 23 selected parameters were analysed.

The pH value in raw leachates from both landfills in all series ranged from 7.8 to 9.1. After treatment, a slight increase to 9.5 was observed in leachates from Legnica (*C. demersum*). Leachates from both landfills were alkaline in nature, which is typical for leachates from mature facilities (i.e., operating >10 years) [48].

The concentrations of heavy metals in leachates from both landfills remained very low throughout the study period, which may be related to the high leachate pH that leads to immobilization of metals by reducing their solubility [49]. The concentrations of chromium, lead, nickel and cadmium in both landfills did not exceed <1 mg/L. During the two-year study, elevated copper and zinc contents (i.e., >1 mg/L) were recorded in the leachates from both landfills, but during the last series of studies (June 2020), the contents of all heavy metals were lower and did not exceed the value of 1 mg/L. According to Naveen et al. [50], the low concentrations of heavy metals in leachate are a confirmation that mainly municipal waste was deposited in the landfill.

Higher concentrations of AN and ON were observed in leachates from the active landfill in Legnica. This could be related to the age of the landfills and their phase of operation, i.e., Legnica—active, Bielawa—closed [32]. AN belongs to the main form of nitrogen found in leachates from landfills [51], which was confirmed by the analyses performed.

Similar contents of sodium, magnesium, calcium and potassium were recorded in leachates from both landfills. The presence of these cations in the leachates may indicate that plant residues and other bio-waste were deposited in the landfills [52].

As the composition of leachates depends on a number of factors and changes over time [3], the last batch performed in June 2020 was compared with previously conducted analyses to verify that the physicochemical composition did not deviate from previous results.

It can be observed that the majority of parameters of the physicochemical composition of leachates from both landfills had values close to the lower limit of the range corresponding to the previously conducted analyses. In the case of the Bielawa landfill, some of the parameters were even below the minimum values (EC, COD, TS, TDS, iron). The biggest difference was observed in the case of COD (a value almost three times lower than previously recorded). The COD content in leachates changes in time and is determined by transformations occurring in the landfill [53,54]. 

In the case of leachate from Legnica in June 2020, the potassium concentration exceeded the maximum value from 2018–2019, while the EC value and iron concentration were lower than the minimum value. There were also significant differences between the composition of leachates from Legnica and Bielawa. This could be related to the age of the landfills and their phase of operation, i.e., Legnica—active, Bielawa—closed [3,55].

Samples collected in June 2020 were treated with *P. australis* and *C. demersum*. It can be observed that for most of the analysed parameters, a reduction was recorded after treatment, but for some parameters an increase was recorded compared to the raw leachate samples. 

Liang et al. [56] proved that *P. australis* and other plants are able to remove between 40 and 80% of organic substances such as COD and AN from landfill leachate. In the study conducted, both plants showed good efficiencies in COD removal, according to the data reported in the literature it was in the range of 40 to 80%, while for AN even a reduction close to 100% was obtained.

Table 3 presents the results of physicochemical analyses of selected parameters of activated sludge collected in June 2020 from the Janówek wastewater treatment plant in Wrocław.

The pH of activated sludge was 7.6 (slightly alkaline reaction). The pH is an important parameter that affects the growth rate and enzymatic activity of activated sludge. Most bacteria thrive at a pH between 4 and 9. It has been shown that its changes can have a significant effect on the abundance of individual species in the population [57].

Furthermore, EC can influence the composition of the bacterial biocenosis of activated sludge. It has been shown that with an increased EC value, nitrification processes are inhibited because nitrifiers are very sensitive to increasing salinity, unlike ammonifiers [58].

The contents of ammonia nitrogen (N-NH_4_^+^), nitrite nitrogen (N-NO_2_^−^) and nitrate nitrogen (N-NO_3_^−^) were low, which may indicate that they were used in the growth processes of activated sludge microorganisms.

### 3.2. Toxicity of Landfill Leachate—Tests on Freshwater Crustacean Daphnia magna Straus

In many countries, including Poland, leachate water quality monitoring is based solely on physicochemical analyses [59]. The studies conducted allow for the identification of many contaminants present in leachate, but some may not be detected. Therefore, toxicity tests, which are conducted on various organisms, can be used to complement the physicochemical analyses. One of the most commonly used in leachate toxicity tests is the crustacean *D. magna* [40,60].

Table 4 presents the results of acute toxicity tests on *D. magna* organisms conducted in the period 2018–2020 (in raw leachate) and after treatment with *P. australis* and *C. demersum* (June 2020 series) at the active landfill in Legnica and the non-operational landfill in Bielawa.

Throughout the study period, higher TU (toxicity units) values, both in raw leachate and after treatment (*C. demersum* and *P. australis*), were recorded for the active landfill in Legnica. 

Most of the values obtained were within the range corresponding to acute toxicity (1.0 < TU < 10), except for two series of tests on leachates from Legnica, where higher values were observed, indicating high acute toxicity, which may be due to a number of reasons.

One possible reason could be the high AN content in leachate from the Legnica landfill, which is indicated in the literature as one of the main causes of elevated toxicity [61]. The maximum values of AN in leachates from Legnica reached 786.1 mg/dm^3^. Heavy metals are considered as another cause of elevated toxicity [39]. However, only in the case of Cu, Zn higher values were recorded (i.e., >1 mg/dm^3^), while the contents of other metals remained at a low level (i.e., < 1 mg/dm^3^) throughout the study period.

In the case of leachates from Bielawa, an exception was the last conducted series of tests (June 2020), when more than twice lower TU values than those observed earlier were obtained. During the testing of leachates from Bielawa in June 2020, very low AN (28.4 mg/dm^3^) and heavy metals (<0.2 mg/dm^3^) were recorded, which may have influenced the slight toxicity of the leachates.

After the applied biological treatment (*C. demersum* and *P. australis*) in the leachates from Legnica, a decrease in TU values was recorded in both cases. Better purification effects were obtained for *C. demersum*, but they still caused acute toxicity (TU > 1) for test organisms. The Bielawa effluent before and after treatment showed negligible acute toxicity for *D.magna* organisms. After treatment with *P. australis* a slight decrease of TU value was observed, while after treatment with *C. demersum* its slight increase was observed. The observed increase could be due to the increased organic matter content, which could lead to acute stress and toxic reactions in the test organisms [30,62].

### 3.3. Analysis of Bacterial Biocenosis Composition in the Samples Studied

**Figure 3:**
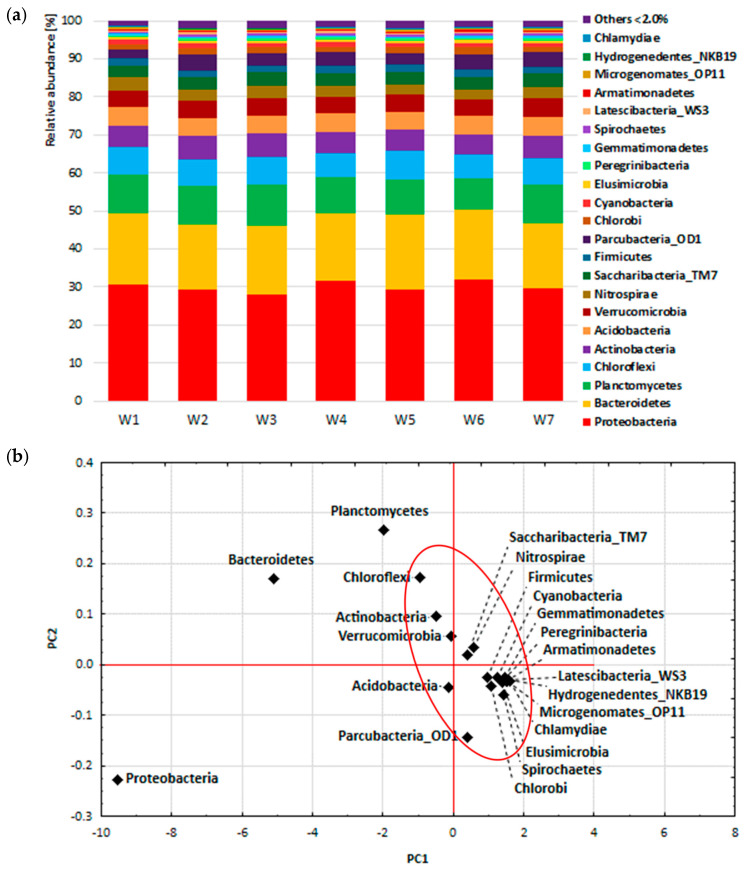
Percentage composition of the bacterial biocenosis of activated sludge (**a**) and projections of cases on the factor plane at the phylum level (**b**).

**Figure 4:**
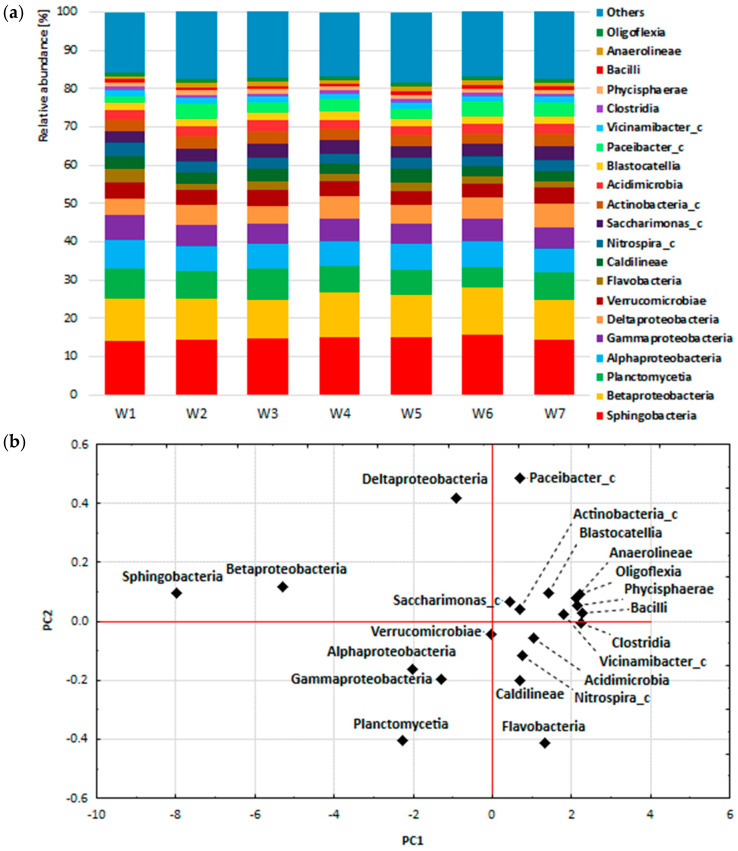
Percentage composition of the bacterial biocenosis of the activated sludge (**a**) and projections of cases on the factor plane at the class level (**b**).

**Figure 5:**
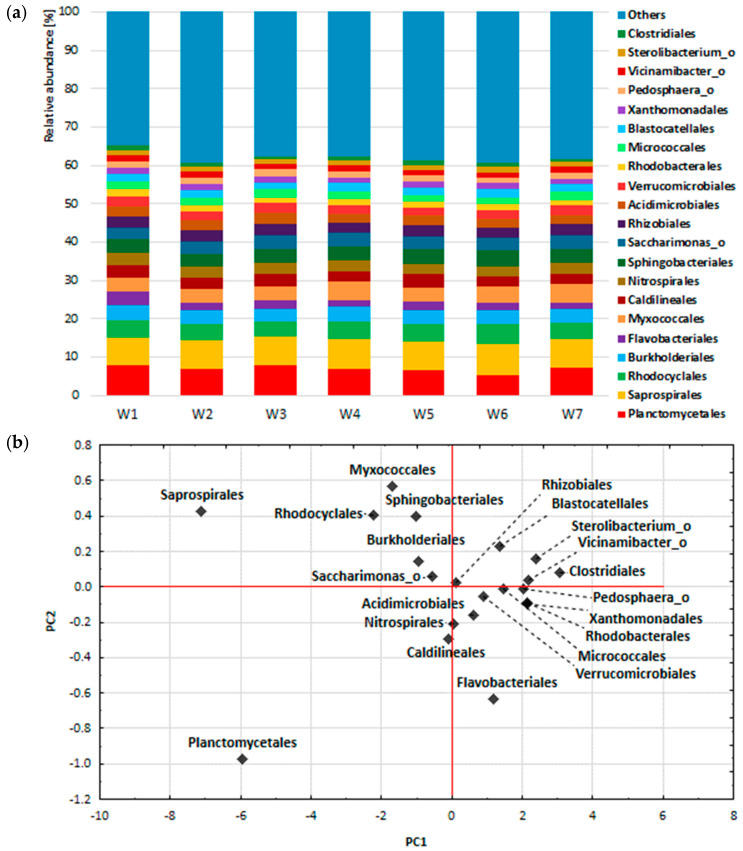
Percentage composition of bacterial biocenosis of activated sludge (**a**) and projections of cases on the factor plane on order level (**b**).

In order to study the general variability of the composition of bacterial communities in the seven analysed samples, the composition of bacterial biocenosis was summed up and the analysis of main components was performed at the phylum, class and order level. The tested samples comprised: mixtures of activated sludge and raw leachates (Legnica leachate—W1, Bielawa leachate—W2), mixtures of activated sludge and leachates treated with *P. australis* (Legnica leachate—W3, Bielawa leachate—W4), mixtures of activated sludge and leachates treated with *C. demersum* (Legnica leachate—W5, Bielawa leachate—W6) and activated sludge (W7). The graphs show the percentages of the most abundant phylum, class and order (Figure 3a, Figure 4a and Figure 5a) and the results of principal component analysis, in the form of projections of cases on the PC1 and PC2 component plane.

NGS sequencing identified 64 phylum, 144 class and 308 order bacteria. Figure 3a shows the composition of the bacterial biocenosis at the phylum level for samples W1–W7. The dominant phylum (in all samples) was Proteobacteria, accounting for between 28% and 31.9% of all bacterial sequences. Bacteroidetes, Planctomycetes, Chloroflexi and Actinobacteria were the minor groups, comprising respectively: Bacteroidetes 17–19.9%, Plantomycetes 8.1–10.8%, Chloroflexi 6.2–7.6% and Actinobacteria 5.3–6.2% of all bacterial sequences. These five types represented between approximately 69.6% and 72.5% of all bacterial types detected in the seven samples. The results of principal component analysis (PCA) confirmed the results of the biocenosis composition analysis. The first principal component explained 99.69% of the total variability of the results and was similarly correlated with all variables (samples W1–W7, correlation coefficient from −0.997 to −0.999). The diagram showing the projection of cases on the PC1-PC2 component plane (Figure 3b) shows the largest contribution with respect to variance of the PC1 factor axis of types: Proteobacteria, Bacteroidetes and Planctomycetes, which together accounted for more than 50% of all bacterial sequences. In contrast, most of the other bacterial types formed a cluster, located near the centre of the PC1 axis (0 values), i.e., they had no significant effect on the magnitude of the variance of this axis.

Figure 4a shows the composition of the bacterial biocenosis in the W1–W7 samples at the class level. The most numerous classes were Sphingobacteriia > Betaproteobacteria > Planctomycetia > Alphaproteobacteria > Gammaproteobacteria. These included: 14.1–15.6% (Sphingobacteria), 10.1–12.3% (Betaproteobacteria), 5.5–7.9% (Planctomycetia), 6.2–7.2% (Alphaproteobacteria) and 5.3–6.7% (Gammaproteobacteria) of all bacterial sequences. Together, these classes accounted for between 43.6% and 47% of the bacterial classes in samples W1–W7. In addition, Betaproteobacteria was the most abundant class in the Proteobacteria type (about 36.6% of sequences). This was followed by Alphaproteobacteria (about 21.9% of sequences), Gammaproteobacteria (about 19.4% of sequences) and Deltaproteobacteria (about 17% of sequences). In contrast, Sphingobacteria was the most abundant class in the Bacteroidetes (about 81% of the sequences). They were followed by: Flavobacteria (about 11.9% of sequences) and Bacteroidia (about 4.2% of sequences) (Figure 4a). At the class level, the results of principal component analysis (PCA) also confirmed the results of the biocenosis composition analysis. The first principal component explained 98.77% of the total variation in the results and was similarly correlated with all variables (samples W1–W7, correlation coefficient from −0.994 to −0.998). Sphingobacteria and Betaproteobacteria made the largest contributions to the variance of the PC1 factorial axis, together accounting for 24.9% to 27.9% of all bacterial sequences. The influence of the other classes on the magnitude of the variance of the PC1 factor axis was much smaller (Figure 4b).

However, the most abundant orders (order) were Planctomycetales > Saprospirales > Rhodocyclales > Burkholderiales > Flavobacteriales (Figure 5a). In spite of the differences in the origin of the leachate samples (operational and closed landfill) and in the methods of treatment (*P. australis* and *C. demersum*), it was observed that the bacterial communities of activated sludge presented a common core, which consisted of 183 orders of bacteria. PCA, conducted at the order level, showed that the first principal component explained 98.77% of the total variance in the results and was similarly correlated with all variables (samples W1–W7, correlation coefficient from −0.968 to −0.997). Saprospirales and Planctomycetales made the largest contributions to the variance of the PC1 factorial axis, together accounting for 13.51% to 15.33% of all bacterial sequences. The effect of the other orders on the magnitude of the variance of the PC1 factorial axis was much smaller, similar to the analysis performed for the classes (Figure 5b).

The study showed that the composition of the bacterial biocenosis at higher taxonomic levels showed little variation between the samples studied (Figure 3a, Figure 4a and Figure 5a). In each sample analysed, Proteobacteria was the most abundant type, which was also found in previous studies conducted on urban wastewater treatment plants [15,20,63,64]. Proteobacteria play a very important role in wastewater treatment by removing organic pollutants, nitrogen and phosphorus [15,65].

The next type in terms of abundance was Bacteroidetes, which was also indicated by other authors as one of the most abundant bacterial types found in sludge. Bacteroidetes may play an important role in wastewater, although it is a less abundant type than Proteobacteria [63]. Bacteria belonging to this type are anaerobes, involved in the degradation of sugars, including glucose and N-acetylglucosamine, and may participate in the conversion of lipopolysaccharides and peptidoglycans released by decomposing cells [64].

Further bacterial types present in the studied samples include Plantomycetes > Chloroflexi > Actinobacteria, while in most studies, in addition to Proteobacteria and Bacteroidetes, Firmicutes and Actinobacteria were among the dominant types of all bacterial sequences [66]. These communities include taxa involved in different metabolic pathways (nitrogen fixation, nitrification, denitrification, sulphur oxidation, etc.) and different physiological groups such as anaerobes, aerobes, phototrophs and heterotrophs, etc. 

Although the bacterial biocenosis at the Phylum level showed little variability between the samples studied, several types of bacteria were detected that were not present in the activated sludge from the treatment plant (sample W7), but appeared after the addition of landfill leachate. These 12 types include: Chrysiogenetes, Lentisphaerae, Rhodothermaeota, Bacteria_uc, Marinimicrobia_SAR406.

Bacterial types that were present in the activated sludge but not observed after the addition of leachate were also observed, among them: Deinococcus-Thermus, Caldiserica, Thermotogae, Aminicenantes_OP8, Deferribacteres.

The most numerous classes were (Figure 2b) Sphingobacteriia > Betaproteobacteria > Planctomycetia > Alphaproteobacteria > Gammaproteobacteria. Additionally, in other studies, Betaproteobacteria constituted one of the more abundant classes, which are mainly involved in AN oxidation and organic matter degradation and the S cycle [20,67].

In the case of the order analysis of the bacterial biocenosis composition, there was not much variation among the seven samples analysed. The most abundant genera in all samples were Planctomycetales > Saprospirales > Rhodocyclales > Burkholderiales > Flavobacteriales (Figure 3c). Analysing the data obtained by other authors [20], it is possible to observe a high variability of bacterial communities at the order level between individual WWTPs, which may result from the fact that the composition of the biocenosis depends on the composition of the inflowing wastewater and the operating conditions of the WWTP; moreover, these are open systems that allow rapid succession of microorganisms.

A total of 734 families were identified in the samples, among which 364 families were common to all analysed samples. The dominant families, found in all seven samples, include: Saprospiraceae, Plactomycetaceae, Comamonadaceae, Nitrospiraceae and Falvobacteriaceae.

A total of 1960 genus were identified, of which 785 were common to all samples analysed. The dominant genus, present in all samples, include: Nitrospira, Dechloromonas, Falvobacterium and Saprospiraceae. 

A total of 4293 species were identified, of which 1254 were common to all samples analysed. The most abundant species recorded in all samples were Nistrospira defluvii group, HQ010811_s, AB186887_s, and Dechloromonas denitrificans group. In the samples that contained leachates from the landfill in Legnica (i.e., W1, W3 and W5), bacteria of the Flavobacterium luticocti species were observed, which were not detected in other samples. The highest number of bacteria of this species was observed in sample W1 (activated sludge mixed with 100% raw leachate from Legnica), while in the other two samples after treatment with *P. australis* and *C. demersum* their content decreased. The species Flavobacterium luticocti includes gram-negative, immobile bacteria that do not form spores. In biochemical tests (API 20NE), Flavobacterium luticocti was positive for nitrate reduction to nitrite, denitrification or d-glucose fermentation, among others, while it was negative in all biochemical tests for carbohydrate metabolism (API 50CH). During research on Flavobacterium luticocti, it was found that it may play an important role in wastewater treatment, but at this moment it is not yet thoroughly investigated [68].

Landfill leachate regardless of the origin of the samples (active and closed landfill) and the method of biological treatment (*P. australis* and *C. demersum*) had no significant effect on the composition of the bacterial biocenosis of the activated sludge. While Barbusinski et al. in their study on the effect of landfill leachate in Poland showed their significant effect on the composition of the activated sludge biocenosis, observing a decrease in the number of filamentous bacteria [69].

### 3.4. Analysis of the Status of Activated Sludge Bacteria by Fluorescence Staining

Mixtures of activated sludge with landfill leachate before and after treatment, as well as the activated sludge itself, were stained with a mixture of SYTO9 and PI dyes and the cells were then observed using a CLSM (confocal laser scanning microscopy) Nikon Eclipse Ni-E C2 (Japan) equipped with 5-megapixel colour digital camera (DS-Fi1c).

**Figure 6:**
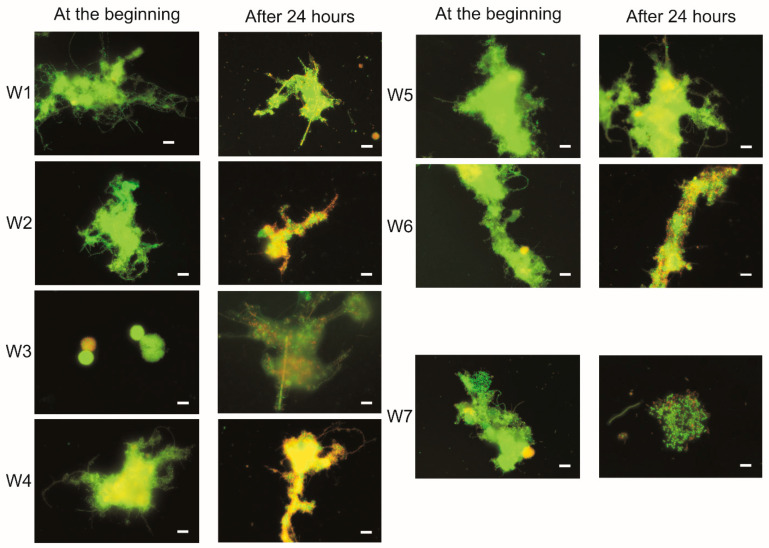
Results of Live/DEAD staining of activated sludge (W7) with leachate samples: raw (Legnica W1, Bielawa W2), after treatment with *P. australis* (Legnica W3, Bielawa W4), after treatment with *C. demersum* (Legnica W5, Bielawa W6). Pictures were taken at the beginning of the experiment and after 24 h. Strip length 10 μm.

For each sample, 10 images were taken at the beginning of the experiment and after 24 h, then the percentage of green and red/yellow surface was calculated. Figure 6 shows selected (out of 10 taken for each sample) LIVE/DEAD staining results of activated sludge with raw leachate samples, treated leachate samples, and activated sludge without additive (W7).

According to the Live/Dead^®^ methodology, bacteria with damaged cell membranes are coloured red, while undamaged cells are coloured green [45].

It can be observed that damaged filamentous bacteria (W2, W4 and W6) were mainly arranged on the outside of the flocs, while undamaged bacteria remained in the flocs, which was also observed by other authors during their study [70].

In the photographs after 24 h of the experiment, an increase in the minor aggregation of activated sludge cells was observed in most samples, except for sample W3 and W7. Furthermore others have also observed increased cell aggregation, which may be a consequence of protection from environmental stresses [71] or cell lysis, attributed to increased DNA release under stress conditions or loss of viability [72].

In all samples analysed, bacteria were mainly stained green, indicating that raw and post-treatment leachates did not significantly affect bacterial cell membranes. 

In the samples in which leachates from the landfill in Bielawa (W2, W4 and W6) were dosed to the sludge, an increase in the number of red-stained bacteria was observed after 24 h of exposure, which indicates damage to cell membranes. Comparing the ratio of green to red lighted area, an increase in the red surface after 24 h was observed by 32 and 33%, respectively, for the samples W2 and W4. This may indicate damage to the cell membranes, although for the sample without the addition of leachates (W7) similar values were observed at the level (35%). 

However, in the case of the second landfill in Legnica (samples W1 and W5), an improvement in the condition of activated sludge was observed (increase of green area) after 24 h of the experiment, which could be explained by supplying leachate to the sludge that contained significant concentrations of nitrogen compounds, including AN of 188.2 mg/L. 

High concentrations of COD or chlorides from landfill leachate did not visibly affect the biocenosis of the activated sludge. Perhaps with a longer escapement time for leachate, greater differences could be observed. There was also no significant fragmentation of the sludge or decrease in the number of filamentous bacteria. It should be noted that monitoring the phenomenon of leachate impact on the condition of the activated sludge biocenosis is difficult and the available methods, although advanced, do not provide full information on the scale of the problem. Domańska et al. [73] suggest to analyse the properties of the wastewater discharged from the treatment plant (outflow) instead of the activated sludge.

### 3.5. Occurrence of Nitrifying Bacteria in Analysed Samples

The basic process of biological nitrogen removal in WWTPs is nitrification, which was conventionally classified as a two-stage process. The first stage is carried out by AOB bacteria, which convert ammonia to nitrite, and then NOB bacteria convert nitrite to nitrate [16]. 

**Figure 7:**
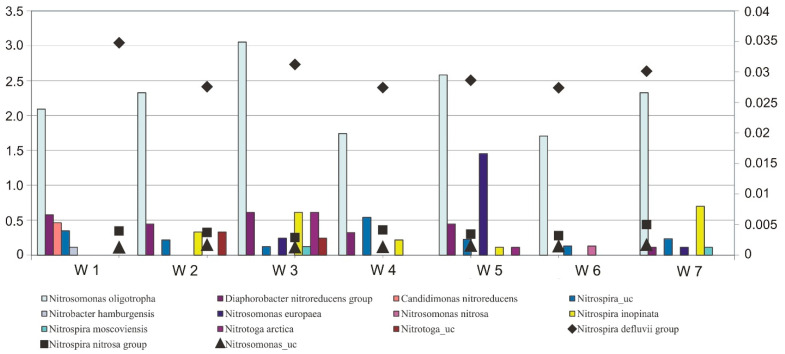
Analysis of the content of nitrifying bacteria in samples of activated sludge (W7) and mixtures with leachates: raw (Legnica W1, Bielawa W2), after treatment with *P. australis* (Legnica W3, Bielawa W4), after treatment with *C. demersum* (Legnica W5, Bielawa W6).

The composition of nitrifying bacteria present in samples of activated sludge and sludge/landfill leachate mixture was analysed. It is considered that nitrifiers are rather sensitive to high salinity [58], although it is possible to distinguish also those that show resistance such as: Nitrosomonas europaea or Nitrobacter_sp [74]. However, this phenomenon negatively affects the biodiversity of microorganisms [75], causing elimination of the so-called salt-intolerant species [76]. Almost almost the same composition of nitrifiers was observed, both in reactors with activated sludge and with mixtures of activated sludge and leachate (Figure 7). 

All samples were dominated by AOB bacteria of the genus Nitrospira, species: Nitrospira defulvii group and Nitrospira nitrosa group, as well as bacteria of the species Nitrosomonas_ uc and Nitrosomonas oligotropha. 

In spite of the fact that almost the same composition of nitrifiers was observed in the analysed samples, differences can be noticed between reactor W1 (activated sludge and raw leachate from Legnica) and the other samples. In sample W1, the appearance of two bacterial species that were not recorded in the other samples was observed, viz: Candidimonas nitroreducens and Nitrobacter hamburgensis.

Candidimonas nitroreducens was first isolated from wastewater sludge compost [77], belongs to the family Alcaligenaceae, and has a nitrate-reducing role [78]. On the other hand, N. hamburgensis oxidizes nitrite to nitrate, moreover, it exhibits resistance to heavy metals and is thought to carry out catabolism pathways of aromatic, organic and monocarbon compounds [79].

In addition, the appearance of bacteria that were not present in the activated sludge sample (W7) was observed in the leachate samples. Nitrosomonas nitrosa appeared in sample W6, Nitrosa arctica appeared in samples W3 and W5, and Nitrotoga_uc appeared in samples W2 and W3.

It was also observed that the bacteria that were present in the activated sludge reactor (W7) were not present in samples W1 and W6. These include the bacteria Nitrospira inopinata, which was the first species of the genus Nitrospira to be discovered, belonging to the fully nitrifying bacteria that carry out both stages of nitrification [18].

Bacteria present in leachate may contribute to the condition of activated sludge, which may have a positive impact on the process and efficiency of treatment in municipal wastewater treatment plants. However, this requires a more extensive study over a longer period of time and an extension of the existing monitoring to assess the composition of the bacterial biocenosis in the landfill. 

The novelty of this study is to investigate the effect of landfill leachate on sludge bacterial biocenosis, which has not been done before. 

The research on the biocenosis of activated sludge and landfill leachate should be expanded and continued, which would contribute to the improvement of leachate treatment technologies. 

## 4. Conclusions

The physicochemical properties of the analysed landfill leachates did not indicate a high degree of contamination; however, throughout the study period they were characterised by quite high toxicity, which may cause adverse effects on living organisms.

The study performed showed that the sources of the samples (active and closed landfill) and the method of treatment (*P. australis* and *C. demersum*) did not significantly affect the composition of the bacterial biocenosis of the activated sludge at higher taxonomic levels (type, class, genus). However, at the species level, the greatest differences were observed in the reactors with activated sludge and leachate from the landfill in Legnica, where bacteria were observed that were not present in any other samples, viz: Flavobacterium luticocti and nitrifying bacteria, viz: Candidimonas nitroreducens and Nitrobacter hamburgensis. 

Furthermore, the results of LIVE/DEAD fluorescence staining showed that the addition of leachate (raw and after treatment) did not significantly affect the cell membranes of activated sludge bacteria. Even a slight improvement in the condition of the sludge was observed after mixing with leachate from Legnica. 

The presence of bacteria that can contribute to improving the condition of sludge may have a beneficial effect on the course and efficiency of treatment in municipal wastewater treatment plants. However, this requires extending the existing monitoring to assess the composition of the bacterial biocoenosis in the landfill and adjusting the further treatment of leachate accordingly.

## Figures and Tables

**Table 1:** List of tested physicochemical parameters with methods of their determination [32].

Pollution Indicators	Name of the Method	Standard/Source
pH	Potentiometric method	ISO 10523:2008
EC	Conductometric method	ISO 7888:1985
COD	Bichromate titration method	ISO 6060:1989
TKN	Method after mineralization with selenium	ISO 5663:1984
ON	Computational method	[33]
AN	Spectrophotometric method	ISO 7150-1:1984
N-NO_3_^−^	Spectrophotometric method	ISO 7890-3:1988
N-NO_2_^−^	Spectrophotometric method	ISO 6777:1984
TS	Computational method	[34]
TDS, TSS	Filtration though glass-fibre filters	ISO 11923:1997
Sulphates	Gravimetric method using barium chloride	ISO 9280:1990
Chlorides	Mohr’s method	ISO 9297:1989
Sodium	Atomic Absorption Spectrometric method (AAS)	ISO 9964-1:1993
Potassium	Atomic Absorption Spectrometric method (AAS)	ISO 9964-2:1993
Calcium, Magnesium	Atomic Absorption Spectrometric method (AAS)	ISO 7980:1986
Iron	Spectrophotometric method	ISO 6332:1988
Manganese	Spectrophotometric method	ISO 6333:1986
Copper, Zinc, Chromium, Lead, Nickel, Cadmium	Atomic Absorption Spectrometric method (AAS)	ISO 15586:2003

**Table 2:** Physicochemical properties of raw leachates from landfills in Bielawa and Legnica, studies conducted in 2018–2020.

Landfill	Unit	Bielawa					Legnica				
		Raw Leachate			After Biological Treatment		Raw Leachate			After Biological Treatment	
Pollution Indicators		Min.	Max.	June 2020	*P. australis*	*C. demersum*	min.	Max.	June 2020	*P. australis*	*C. demersum*
**pH**	-	7.8	9.1	8.4	9.1	9.4	8.0	8.9	8.8	9.1	9.5
**EC**	μS/cm	3048.0	5075.0	2318.0	2340.0	2419.0	8417.0	11370.0	7791.0	8109.0	8503.0
**COD**	mg O_2_/dm^3^	954.0	4270.0	321.8	154.0	198.8	1585.0	3800.0	2007.0	1577.0	1520.8
**TKN**	mg N/dm^3^	32.2	294.5	51.1	6.2	11.9	167.6	907.4	269.9	30.4	58.2
**ON**	mg N_org_/dm^3^	14.6	182.8	22.7	6.0	11.9	4.5	121.3	81.7	6.8	58.2
**AN**	mg N_NH4_/dm^3^	17.6	231.2	28.4	0.2	0.0	66.1	786.1	188.2	23.6	0.0
**TS**	mg/dm^3^	2580.0	8745.0	2045.0	2028.0	2196.0	6210.0	8245.0	7395.0	7669.0	8545.0
**TDS**	mg/dm^3^	2140.0	3050.0	1920.0	1864.0	2178.0	6195.0	7830.0	7065.0	7613.0	7697.0
**TSS**	mg/dm^3^	105.0	5995.0	125.0	23.0	639.0	15.0	1870.0	330.0	168.0	320.0
**Sulfates**	mg SO_4_/dm^3^	139.1	1884.0	1481.0	268.2	207.3	80.6	396.6	261.6	477.2	419.6
**Chlorides**	mg Cl/dm^3^	5.5	765.0	303.0	670.0	468.0	22.0	2811.0	2160.0	2330.0	2920.0
**Sodium**	mg Na/dm^3^	132.3	285.8	151.8	162.9	307.5	175.2	329.2	177.8	809.5	876.5
**Potassium**	mg K/dm^3^	188.8	265.6	256.2	207.4	368.5	238.2	317.2	507.6	997.0	1025.0
**Calcium**	mg Ca/dm^3^	69.5	194.3	150.3	188.5	60.9	43.9	113.8	68.1	113.1	91.1
**Magnese**	mg Mg/dm^3^	61.3	133.4	79.3	72.3	67.0	70.2	133.8	87.9	90.6	92.1
**Iron**	mg Fe/dm^3^	1.6	18.0	0.5	0.2	0.2	2.6	10.6	2.2	2.1	1.1
**Manganese**	mg Mn/dm^3^	0.1	2.4	0.4	0.1	0.1	0.2	0.6	0.5	0.5	0.5
**Copper**	mg Cu/dm^3^	0.0	4.7	0.0	0.0	0.0	0.1	4.0	0.1	0.1	0.1
**Zinc**	mg Zn/dm^3^	0.3	1.6	0.2	0.2	0.2	0.1	2.0	0.5	0.3	0.8
**Chromium**	mg Cr/dm^3^	0.0	0.5	0.0	0.0	0.0	0.0	0.6	0.2	0.0	0.2
**Lead**	mg Pb/dm^3^	0.0	0.2	0.0	0.0	0.0	0.0	0.3	0.1	0.0	0.1
**Nickel**	mg Ni/dm^3^	0.0	0.1	0.0	0.1	0.1	0.1	0.4	0.2	0.1	0.2
**Cadmium**	mg Cd/dm^3^	0.0	0.0	0.0	0.0	0.0	0.0	0.0	0.0	0.0	0.0

**Table 3:** Selected properties of activated sludge from wastewater treatment plant in Wrocław.

Pollution Indicators.	Unit	June 20
**pH** (**range**)	-	7.6
**EC**	mS/ cm	1330
**AN**	mg N-NH_4_^+^/ dm^3^	0.070
**Nitrite nitrogen**	mg N-NO_2_^−^/dm^3^	0.015
**Nitrate nitrogen**	mg N-NO_3_^−^/dm^3^	0.097

**Table 4:** TU values in the raw landfill leachate (series 2018–2020) and series June 2020 before and after the biological treatment with *Phragmites australis* and *Ceratophyllum demersum* L.

Research Object	Raw Leachate			Series June 2020 after Biological Treatment	
	Min. Values (2018–2019)	Max. Values (2018–2019)	June 2020 Series	*Ceratophyllum demersum*	*Phragmites australis*
**Bielawa**	1.58	3.71	0.64	0.92	0.51
**Legnica**	3.19	31.25	3.37	2.31	2.73

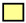
 negligible acute toxicity: 0.4 < TU < 1, 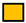 acute toxicity: 1.0 < TU < 10, 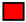 high acute toxicity: 10 < TU < 100 [41].

## Data Availability

Not applicable.
